# Adverse childhood experiences increase the risk for eating disorders among adolescents

**DOI:** 10.3389/fpsyg.2022.1063693

**Published:** 2022-12-12

**Authors:** Beáta Kovács-Tóth, Barnabás Oláh, Ildikó Kuritárné Szabó, Ferenc Túry

**Affiliations:** ^1^Institute of Behavioural Sciences, Faculty of Medicine, University of Debrecen, Debrecen, Hungary; ^2^Doctoral School of Health Sciences, University of Debrecen, Debrecen, Hungary; ^3^Institute of Behavioural Sciences, Semmelweis University, Budapest, Hungary

**Keywords:** eating disorders, anorexia nervosa, obesity, adverse childhood experiences, childhood maltreatment, family/household dysfunction

## Abstract

**Purpose:**

Traumatic events often feature prominently in eating disorders. A questionnaire survey to assess the relation of eating disorder risk to the frequency of adverse childhood experiences (ACEs) and the possible association of eating disorder risk with a particular type of ACE was conducted in a community sample of Hungarian adolescents.

**Methods:**

Demographic and anthropometric data, risk for eating disorders (by SCOFF questionnaire), and ACEs (by ACE score calculator) were collected from 432 adolescents aged 12–17 years.

**Results:**

Adolescents who had undergone four or more ACEs were 5.7 times more likely to be in the high eating disorder risk group than those who did not report any ACEs. Cumulative maltreatment showed a greater association with overall risk for eating disorders than cumulative family dysfunction. There is an increased risk of eating disorders from emotional maltreatment (*OR* = 3.475), physical maltreatment (*OR* = 3.440), sexual maltreatment (*OR* = 10.973), and emotional neglect (*OR* = 3.331). Dysfunctional family circumstances revealed an association with household mental illness (*OR* = 3.401).

**Conclusion:**

Our study of the connection between eating disorder risk and ACE is the first of its kind in Central and Eastern Europe. Maltreatments had a greater role than family dysfunctions in increasing the risk of eating disorders. Our findings contribute to a more precise understanding of the role that ACEs play in eating disorders. It is important to bring to clinicians’ attention the importance of ACEs in the diagnosis and therapy of eating disorders and their potentially fundamental significance for therapy.

## Introduction

Traumatic events can feature prominently in the background of eating disorders (EDs – anorexia nervosa [AN] and bulimia nervosa [BN]). Several studies have shown the significance of physical, sexual, and psychological abuse, and emotional and physical neglect in adult EDs (Brewerton, 2007; Caslini et al., 2016; Guillaume et al., 2016; Molendijk et al., 2017; Pignatelli et al., 2017; Trottier and MacDonald, 2017; Amianto et al., 2018; Palmisano et al., 2018). Childhood sexual abuse is the most extensively studied form of traumatic experience in relation to EDs, and literature data reveal that it may have role in the development of the AN binge/purge subtype, but not the restrictive subtype (Trottier and MacDonald, 2017; White et al., 2018). In an adolescent outpatient population, 35% of the subjects reported one or more lifetime traumatic events – 10% reported harassment, 9% had a significant death/loss experience, and 8% reported having undergone sexual abuse (White et al., 2018). In a small group of ED patients (*N* = 40) emotional neglect showed the closest correlation with EDs, followed by emotional abuse, physical neglect, physical abuse, and sexual abuse (Brustenghi et al., 2019). Emotional dysregulation may be the mediating factor between childhood abuse and EDs (Racine and Wildes, 2015). ED patients with childhood abuse in their history showed earlier onset of the disorder, more severe psychopathological symptoms, and higher frequency of binge episodes and purging (Trottier and MacDonald, 2017). Traumatization particularly shows up as an aetiological component of subtypes characterized by impulse control disorders, multi-impulsive AN and BN with comorbid borderline personality disorder (Fernandez-Aranda et al., 2008). Impulsive and compulsive tendencies have a mediating role between childhood sexual abuse and EDs (Dworkin et al., 2014). In addition, ACE may influence by in the development of obesity as well (Gustafson and Sarwer, 2004; Hemmingsson et al., 2014).

Adverse childhood experiences (ACEs) can play a significant role in the development of EDs. According to definition, ACE is an extremely complex and multifaceted notion including not only abuse and neglect (maltreatment) affecting a child directly, but also the household harm a child experiences in their family in an indirect manner (family dysfunction). Family dysfunction includes separation/divorce, witnessing violent treatment of mother, household substance abuse, household mental illness, incarcerated household member. Therefore, the notion of ACE extends beyond direct harm (maltreatment) suffered by the child, and includes exposure to indirect harm as well (Felitti et al., 1998).

Childhood and adolescence are key developmental periods for the formation of various risk behaviors, including EDs (Campbell and Peebles, 2014; Pearson et al., 2014). Regarding the ED risk, some European studies based on the SCOFF questionnaire are overviewed in our paper, because in the present study this questionnaire was also used (see the description of this instrument in the Methods section). In Western European studies using the SCOFF, the risk of EDs was found to be 21.9–29.4% among 10–18 year old girls, and around half of these scores among boys (18, 19, 20). Comparing Western and Eastern European settings, it is to be mentioned that in a SCOFF-based study of an adolescent population in Hungary (mean age: 16.62 years), a prevalence of 36.8 and 15.8% was found among girls and boys, respectively (Horvath et al., 2020). As for the global prevalence of DSM-5 EDs in Western settings, 5.5–17.9% of young women and 0.6–2.4% of young men have experienced a DSM-5 eating disorder by early adulthood (Silen and Keski-Rahkonen, 2022).

Several epidemiological studies have been carried out in Central and Eastern Europe to detect EDs in teenagers with the help of instruments other than SCOFF. A survey of 14–16-year-old Polish schoolgirls that used the 26-item version of the Eating Attitudes Test (EAT) found no AN or BN and a 2.34% prevalence of subclinical disorders (Wlodarczyk-Bisaga and Dolan, 1996). In Bulgaria, the rate of positive EAT scores among schoolgirls was 10.4% (Boyadjieva and Steinhausen, 1996). In Croatia, the positive EAT rate was 10.3% among girls aged 10–15 and 7.6% among those aged 14–18 (Ambrosi-Randić and Pokrajac-Bulian, 2005). Among Romanian schoolgirls, the EAT found prevalence of 0.3% for AN and 0.8% for BN, and 1.8% for subclinical AN and 1.4% for subclinical BN (Kovács and Szabó, 2009). In a representative questionnaire survey in Hungary using the Eating Disorder Inventory (EDI), the point prevalences of AN and BN for women aged 15–24 were 0.03 and 0.4% respectively, and subclinical AN and BN had a score of 1.1 and 1.5%, respectively (Szumska et al., 2005).

A comparison of Eastern and Western Europe by Podar and Allik (2009) involving a meta-analysis of 94 studies found that non-Western participants scored higher than western participants on most of the EDI subscales, both in general population and ED samples.

Research on the links between EDs and traumatization initially concentrated on surveying emotional, physical and sexual maltreatment, and then included the role of relationship violence. The next major wave of research examined the possible factors linking traumatic experiences to EDs, including psychological (e.g., emotion dysregulation, anger, dissociative experience, impulsivity and compulsivity) and neurobiological factors (Trottier and MacDonald, 2017).

There are no data available on the association of ACEs with risk of EDs in adolescents. No investigation has been carried out using a screening test that embraces dysfunctional family circumstances and is thus capable of measuring indirect in addition to direct harm.

### Aims of the present study

1. To determine the frequency of ED risk occurrence in this young population, with special focus on gender differences.
2. To assess the relationship between the accumulation of ACE experiences and ED risk. Our hypothesis is that the higher the number of ACE experiences, the greater the ED risk.
3. To study the change in ED risk by breaking down ACE harm into direct and indirect harm and thus determine whether an accumulation of maltreatment or dysfunctional family circumstances are associated with greater ED risk.
4. To determine whether any particular ACE type is associated with ED risk.
5. To examine the gender differences in relation to the effect of ACE on the ED risk.
6. To examine the associations between risk for EDs and body mass index (BMI) categories.
7. To determine how ACEs are related to risk for AN and obesity.

## Materials and methods

### Sampling and data collection

A cross-sectional retrospective study was conducted in a community sample of Hungarian adolescents. Data collection from an anonymous self-report questionnaire was carried out between 2018 and 2020 in schools in four counties. We contacted five schools in four cities and visited grade 6–11 students whose age ranged from 12 to 17 years. Data collection was performed in groups, under the assistance of health psychology students. We contacted 535 adolescents. Parental informed consent rate was 99.4%. Students’ absenteeism on the date of the data collection further decreased the response rate to 87.1%, thus altogether 466 adolescents were recruited, who also gave their written informed consent. After data cleaning, 32 participants were excluded due to incomplete questionnaire responses. Participants with incomplete information about a SCOFF question were included if they already scored above the threshold (2≤) or when they scored inalterably under the threshold (score was 0, and there was only one response missing). In the final sample 432 participants were included in analyses of ACE experiences and ED risk. A group of 10 people did not provide one or more anthropometric data (weight, height) – this is why analyses involving BMI categories rely on responses from 422 participants. The demographic features of the sample are provided in Table 1. Ethical approval was issued by the Research Ethics Committee of the Hungarian Medical Research Council (file number ETT TUKEB 47848–7/2018/EKU).

**Table 1:** Demographic characteristics of the sample overall and by gender.

Sociodemographic variable	Boys (*N* = 153)	Girls (*N* = 279)	Total (*N* = 432)
Age mean (SD)	14.76 (0.96)	14.71 (1.14)	14.71 (1.08)
**Location *N* (%)**			
Village	42 (27.5)	61 (21.9)	103 (23.8)
Town	54 (35.3)	115 (41.2)	169 (39.1)
City	57 (37.3)	103 (36.9)	160 (37.0)
**Maternal education *N* (%)**			
Primary or less than primary	3 (2.0)	5 (1.8)	8 (1.9)
Secondary	41 (26.8)	94 (33.7)	135 (31.3)
Tertiary	109 (71.2)	180 (64.5)	289 (66.9)

### Measures

Apart from demographic data (gender, age, location, maternal education), the SCOFF questionnaire was extended with anthropometric data (weight, height), and the ACE Score Calculator was employed.

### SCOFF questionnaire

The questionnaire measures ED risk through five questions [(Morgan et al., 1999), Hungarian adaptation: (Dukay-Szabó et al., 2016)]. It is a screening instrument designed to clarify suspicion of an ED, and is not suitable for setting up a diagnosis. The questions can be delivered either verbally or in written form, and they concentrate on self-induced vomiting, losing control of eating, severe weight loss, body image disorder, and food dominance. In Hungarian practice, two “yes” answers are regarded as the threshold for ED risk.

Relating to other ED symptoms, to determine the BMI category (underweight, healthy weight, overweight, obesity) it is necessary to calculate which BMI percentile the individual falls into. This requires data on age, sex, weight, and height. To determine the BMI percentile and category, we used a calculator available from the website of the Centers for Disease Control and Prevention (n.d.).

A score above the cut-offs associated with minimum two “yes” answers and underweight BMI category indicates a high risk of AN.

### Ace score calculator

The ACE Score Calculator is a short self-report questionnaire comprising ten yes-no items (Anda et al., 2010; Ujhelyiné Nagy and Kuritárné Szabó, 2020). Five types of maltreatment (i.e., direct ACEs) are evaluated: emotional, physical, sexual abuse, physical and emotional neglect. In addition, five types of household dysfunction (i.e., indirect ACEs) are also assessed: parental separation/divorce, witnessing violent treatment of mother, household substance abuse, household mental illness, incarcerated household member. The questionnaire describes behavioral patterns. Based on the number of types of ACEs, a cumulative ACE score is calculated between 0 and 10. It is a severity index, which indicates how many types of adversities a person has experienced in their childhood. Item contents and item response options are given in the Appendix.

### Statistical analyses

Statistical analyses were performed using IBM SPSS Statistics v. 23. Frequency statistics were carried out on responses for the SCOFF items, risk for EDs, BMI categories and the accumulation and frequency of ACEs. Logistic regression analyses were conducted to assess the relationship between ACEs and the overall risk for EDs and obesity, and between BMI categories and risk for EDs. No logistic regression could be performed for ACE with risk for AN due to the small number of cases with risk for AN (*n* = 4).

The overall risk for EDs was a binary dependent variable determined with the help of the SCOFF questionnaire (cut-off: 2≤; low risk for any EDs vs. at risk or have that disorder). The independent variables were BMI categories, the number of ACEs (overall, and by childhood maltreatment and family dysfunction), or positive/negative responses for the types of ACEs. All the models were adjusted for age, gender, location, and maternal education to calculate adjusted odds ratios (ORs). We conducted post-test analysis using the adjusted Wald test.

## Results

### Frequency of risk for eating disorders, BMI categories and ACEs

Risk for EDs by the SCOFF was found in 19.2% of the sample, 26.2% of girls and 6.5% of boys (Table 2). A high risk for AN was only found in girls, corresponding to 1.4% of girls, 0.9% of the total sample. In terms of BMI categories, 81.3% of the participants were of healthy weight (*n* = 343), 4.3% were underweight (*n* = 18), 10.2% were overweight (*n* = 43), and 4.3% were obese (*n* = 18).

**Table 2:** Frequency of risk for eating disorders (EDs), BMI categories, and adverse childhood experiences (ACEs) in the sample overall and by gender.

	Boys (*N* = 153)	Girls (*N* = 279)	Total (*N* = 432)	*p*-value^a^
**SCOFF items *N* (%)**				
Purging	2 (1.3)	11 (3.9)	13 (3.0)	0.125
Loss of control	15 (9.8)	106 (38.0)	121 (28.0)	<0.001**
Weight loss	19 (12.4)	36 (12.9)	55 (12.7)	0.885
Body image distortion	10 (6.6)	53 (19.0)	63 (14.6)	<0.001**
Preoccupation with food	8 (5.4)	41 (14.9)	49 (11.5)	0.003*
**Risk for EDs *N* (%)**				
(cut-off: SCOFF score ≥ 2)	10 (6.5)	73 (26.2)	83 (19.2)	<0.001**
Risk for anorexia nervosa	-	4 (1.4)	4 (0.9)	0.137
**BMI category (*N* = 422) *N* (%)**				
Healthy weight	117 (77.5)	226 (83.4)	343 (81.3)	0.085
Underweight	4 (2.6)	14 (5.2)	18 (4.3)	
Overweight	21 (13.9)	22 (8.1)	43 (10.2)	
Obesity	9 (6.0)	9 (3.3)	18 (4.3)	
**Total ACE score *N* (%)**				
0	107 (69.9)	151 (54.1)	258 (59.7)	0.003*
1	27 (17.6)	63 (22.6)	90 (20.8)	
2	15 (9.8)	30 (10.8)	45 (10.4)	
3	2 (1.3)	12 (4.3)	14 (3.2)	
≥4	2 (1.3)	23 (8.2)	25 (5.8)	
**Maltreatment ACE score *N* (%)**				
0	132 (86.3)	204 (73.1)	336 (77.8)	
1	14 (9.2)	43 (15.4)	57 (13.2)	
2	7 (4.6)	19 (6.8)	26 (6.0)	0.023*
3	-	10 (3.6)	10 (2.3)	
4	-	2 (0.7)	2 (0.5)	
5	-	1 (0.4)	1 (0.2)	
**Family dysfunction ACE score *N* (%)**				
0	117 (76.5)	178 (63.8)	295 (68.3)	
1	29 (19.0)	67 (24.0)	96 (22.2)	
2	7 (4.6)	26 (9.3)	33 (7.6)	0.013*
3	-	8 (2.9)	8 (1.9)	
4	-	-	-	
5	-	-	-	
**Type of ACEs**				
*Maltreatment N (%)*				
Emotional abuse	14 (9.2)	43 (15.4)	57 (13.2)	0.066
Physical abuse	9 (5.9)	18 (6.5)	27 (6.3)	0.815
Sexual abuse	1 (0.7)	7 (2.5)	8 (1.9)	0.171
Emotional neglect	4 (2.6)	52 (18.6)	56 (13.0)	<0.001**
Physical neglect	-	4 (1.4)	4 (0.9)	0.137
*Family dysfunction N (%)*				
Parental separation/divorce	30 (19.6)	71 (25.4)	101 (23.4)	0.170
Household physical violence	3 (2.0)	11 (3.9)	14 (3.2)	0.266
Household substance abuse	7 (4.6)	27 (9.7)	34 (7.9)	0.060
Household mental illness	3 (2.0)	29 (10.4)	32 (7.4)	0.001*
Incarcerated household member	-	5 (1.8)	5 (1.2)	0.096

^a^Indicates the application of Pearson’s Chi-squared test on gender differences within population. *p < 0.05, **p < 0.001.

A total of 19.4% of adolescents reported two or more, and 5.8% reported four or more ACEs. As regards ACE score patterning, 9% of the sample reported two or more forms of maltreatment, and 9.5% of the sample reported two or more instances family dysfunction.

The most prevalent forms of reported child maltreatment in the sample were emotional abuse (13.2%) and emotional neglect (13.0%). The most frequent dysfunctional household condition was parental divorce or separation (23.4%).

As regards gender, the Pearson’s chi-squared test indicated that the accumulation of adversities is significantly more frequent among girls: 8.2% reported four or more adversities, and 23.3% reported two or more adverse experiences. Moreover, more than six times as many girls (8.2%) had reported ≥4 adversities compared to boys (1.3%).

Emotional neglect was nearly seven times more prevalent among girls (18.6%) than boys (2.6%), and five times as many girls as boys had a mentally ill family member (girls: 10.4%; boys: 2.0%).

### Associations between risk for eating disorders and the number and types of ACEs

As presented in Table 3, adolescents reporting three ACEs were 3.41 times, persons with four or more ACEs were 5.66 times more likely to be at risk for developing an ED than adolescents without an ACE.

**Table 3:** Adjusted odds ratios for overall risk for eating disorders by the total number of adverse childhood experiences (ACEs) and forms of maltreatment, and by presence of a type of ACE.

	Risk for eating disorders		
	OR	*p*-value	Model
**ACE Score (ref. 0)**			
1	1.595	0.155	
2	1.568	0.296	*χ*^2^ (10) = 52.191 *p* < 0.001
3	3.407	0.040*	
≥4	5.652	<0.001**	
**Maltreatment ACE Score (ref.: 0)**			
1	4.072	<0.001**	*χ*^2^ (11) = 66.256 *p* < 0.001
2	2.719	0.036*	
3	9.337	0.002*	
4	4.650	0.344	
5	NaN	1.000	
**Types of ACEs** (ref.: response to that type is “No”)			
**Maltreatment**			
Emotional abuse	3.475	<0.001**	*χ*^2^ (7) = 50.898 *p* < 0.001
Physical abuse	3.440	0.009*	*χ*^2^ (7) = 43.271 *p* < 0.001
Sexual abuse	10.973	0.006*	*χ*^2^ (7) = 45.851 *p* < 0.001
Emotional neglect	3.331	<0.001*	*χ*^2^ (7) = 50.529 *p* < 0.001
Physical neglect	2.602	0.367	*χ*^2^ (7) = 37.458 *p* < 0.001
**Family dysfunction**			
Parental separation/divorce	0.979	0.944	*χ*^2^ (7) = 36.662 *p* < 0.001
Household physical violence	1.233	0.750	*χ*^2^ (7) = 36.757 *p* < 0.001
Household substance abuse	1.354	0.476	*χ*^2^ (7) = 37.151 *p* < 0.001
Household mental illness	3.401	0.002*	*χ*^2^ (7) = 45.648 *p* < 0.001
Incarcerated household member	1.507	0.660	*χ*^2^ (7) = 36.844 *p* < 0.001

Logistic regression with entry method, adjusted for age, gender, location and maternal education. *p < 0.05; **p < 0.001.

Reported exposure to one (*OR* = 4.072), two (*OR* = 2.719) or three (*OR* = 9.337) forms of childhood maltreatment was significantly associated with increased odds for overall risk for EDs among adolescents, compared to those reporting no maltreatments. The small sample sizes in the 4 (*n* = 2) and 5 (*n* = 1) maltreatment categories limited the possibility of analysing their association with risk for EDs.

Accumulation of family dysfunction was not significantly associated with increased odds for being at risk or having EDs. The small sample size in the upper categories of the number of family dysfunction forms [3 (*n* = 8), 4, 5 (*n* = 0)] limited the evaluation of the risk for ED–ACE association.

Experiencing emotional neglect (*OR* = 3.331), physical abuse (*OR* = 3.440), emotional abuse (*OR* = 3.475), and especially a history of sexual abuse (*OR* = 10.973) highly increased the odds of being at risk for EDs. Reporting the experience of household mental illness (*OR* = 3.401) was also associated with increased odds for overall risk for EDs. We did not find any significant association between other types of ACEs and risk for EDs.

### Associations between risk for eating disorders and BMI categories

Analysis of the associations between BMI categories and risk for EDs showed obese adolescents to be significantly more likely to be at risk of, or to have, an ED (*OR* = 8.872). No associations were found between underweight or overweight adolescents and risk for EDs (Table 4).

**Table 4:** Adjusted odds ratios for risk for eating disorders by BMI categories.

BMI (ref.: normal weight)	Risk for eating disorders			OR	*p*-value	Model
Underweight	1.246	0.719	*χ*^2^ (9) = 51.164 *p* < 0.001
Overweight	1.483	0.389	
Obese	8.872	<0.001**		

Logistic regression with entry method, adjusted for age, gender, location and maternal education. **p < 0.001.

### Associations between obesity and ACEs

Accumulation of childhood maltreatment was significantly associated with increased risk of obesity. Adolescents reported one (*OR* = 4.703) or three (*OR* = 35.029) forms of maltreatment were much more likely to be obese than those who reported no maltreatment (Table 5). We found no significant relationship between obesity and the total ACE score or family dysfunction related ACE score.

**Table 5:** Adjusted odds ratios for obesity by the number of maltreatment forms.

Maltreatment ACE score (ref.: 0)	Obesity			OR	*p*-value	Model
1	4.703	0.018*	*χ*^2^ (11) = 22.560 *p* = 0.020
2	1.887	0.569	
3	35.029	0.002*	
4	0.000	1.000	
5	NaN	0.999		

Logistic regression with entry method, adjusted for age, gender, location, and maternal education. *p < 0.05.

## Discussion

In the 12–17 age group we studied, the SCOFF questionnaire found risk for ED in 19.2–26.2% of girls and 6.5% of boys. This gender difference is well known, and is probably due to the cultural pressure of ideal slimness, which girls are more exposed to. In general, previous studies have found slightly lower prevalence; they, however, have mostly included lower average age participants (Holling and Schlack, 2007; Hudson et al., 2007; Lahteenmaki et al., 2009; Leung et al., 2009; McBride et al., 2013; Esteban-Gonzalo et al., 2014; Solmi et al., 2014; Zeiler et al., 2016). We found one comparable SCOFF figure from Central and Eastern Europe: a 26.4% positive rate among a sample of adolescents of an average age of 16.62 years in Hungary (Horvath et al., 2020). This is further supported by our figure of 19.2% (26.2% of girls and 6.5% of boys), because the mean age in our sample was approximately 2 years lower (14.71). Earlier studies in the region used other instruments, and showed lower levels of ED risk (see Introduction). This difference may be due to methodological peculiarities or differences. The instruments used for these previous studies did not measure binge eating disorder or atypical eating disorders (in DSM-IV: eating disorder not otherwise specified [EDNOS], in DSM-5: other specified feeding or eating disorders [OSFED]). SCOFF probably has a broader scope in identifying ED risk.

ED risk was higher among adolescents who reported more ACEs. Frequent harmful experiences, therefore, play a part in the aetiology of eating disorders. This is confirmed by research into the effects of traumatic experiences [for a review, see (Trottier and MacDonald, 2017)]. Previous studies had not examined the significance of dysfunctional family circumstances.

A breakdown of ACEs into direct and indirect harm revealed that ED risk is more strongly related to maltreatment (direct harm) than family dysfunction (indirect harm). Sexual abuse has a particularly marked pathogenic role, and our findings confirm previous data suggesting the significance of sexual abuse in increasing ED risk (Carter et al., 2006; Trottier and MacDonald, 2017).

Research into the prevalence of sexual abuse has tended to concentrate on girls, which is reflected in the relative abundance of data. Although childhood abuse may also play a role for boys, it is more difficult to detect because males tend to manifest traits of a more externalizing nature (such as aggression). Furthermore, the earlier ages for sexual abuse among boys hamper reliable reporting; and it is more difficult for boys to disclose acts of sexual abuse because of cultural roles that reduce the admission of vulnerability characteristics in males (Thompson and Wonderlich, 2004).

In addition to sexual abuse, our findings show that physical abuse and emotional neglect are significant indicators of ED risk.

One of the gender differences in our study is related to the response to the question of emotional neglect: nine times as many girls as boys responded positively. Our proposed explanation is that girls are more sensitive to emotional attitudes and the associated deficiencies, and demand more and warmer interactions. Girls are also more inclined than boys to talk about the emotional aspects of their experiences, and use more emotional words than boys when talking about frightening events (Fivush et al., 2000). Girls have a greater tendency to reveal/express negative emotions such as sadness and fear (Garside and Klimes-Dougan, 2002). Another significant figure is that nearly five times as many girls as boys reported family members with mental illness, which may be the result of a greater inclination among girls to recognize and/or admit having an ACE.

Emotional dysregulation and dissociation may be mediating factors between childhood maltreatment and EDs (Moulton et al., 2015; Racine and Wildes, 2015). Maltreatment (sexual, physical and emotional) often lies in the background of vulnerable emotional regulation mechanisms. Inappropriate reaction to a child’s emotional state may negatively influence emotional regulation capability. An eating disorder can promote avoidance, distraction or dulling of emotions, and eating may provide a source of pleasure and fill emotional gaps.

The concept of development traumatization disorder (Van der Kolk et al., 2009) offers a useful explanation for the emotional, behavioral and self-regulation difficulties observed among persons with eating disorders. The areas of development where childhood traumatization takes most striking effect are disrupted attachment, biological regulation, emotional and behavioral regulation, cognition and self-concept (Cook et al., 2003). This has been confirmed by empirical research (Fusco and Cahalane, 2013; Teicher et al., 2016).

Traumatization usually appears as a nonspecific risk factor in the history of an eating disorder patient, and may have a circular causal role as part of a complex pathomechanism. If, however, the person experienced trauma in an eating related context, the causal relationship may be tight (such as eating-related parental aggression, coercion and punishment; or bulimic vomiting in consequence of coercion to oral sexual activity).

Control is a central question in eating disorders, and the level of parental control and control-related maltreatment are potentially significant in the development of symptoms. The interaction between perceived parental treatment and childhood traumatization may contribute to interpersonal uncertainty, which is one of the most important dimensions of the psychopathological phenomena of eating disorders (Monteleone et al., 2020).

There is increasing evidence that severe and complex childhood traumatization is involved in the etiology of borderline personality disorder (BPD) (Bandelow et al., 2005; Kuritárné, 2008). The reason for mentioning BPD in the context of this review is that the multi-impulsive subtype of eating disorders is associated with this particular personality disorder.

Obesity has been found to be associated with increased risk for EDs (*OR* = 8.872) and the accumulation of childhood maltreatment. One study found that people with obesity scored higher in emotional and sexual maltreatment and neglect than the healthy control group. Binge eaters in the obese group reported a higher level of maltreatment and neglect than non-bingeing obese persons and healthy controls. The bingeing and obese groups differed in the frequency of physical and sexual maltreatment. The association between childhood traumatic events and adult obesity has been confirmed independently (Trottier and MacDonald, 2017). It is a well-known fact that after sexual abuse, women hide their sexually attractive bodies by becoming obese so as to prevent the recurrence of abuse (Felitti, 1991).

We also examined some dysfunctional family circumstances, of which the most significant for ED risk was the presence of a family member with a mental illness. This may be explained by the common tendency for mental illness to give rise to enmeshment and dysfunctional family boundaries (Minuchin, 1974). Diffuse, enmeshed boundaries are important risk factors for psychosomatic disorders.

We may reasonably surmise that the role of ACEs in EDs forms a continuous spectrum from mild family/environmental stress situations to direct abuse, similarly to spectra widely accepted in psychiatry (addictive spectrum, eating disorder continuum, obsessive compulsive spectrum etc.). Everyday stress plays a well-known part in psychosomatic and other psychiatric disorders (Kuritárné, 2008). Such are enmeshment, overprotectiveness, rigidity, lack of conflict resolution, and the involvement of the child in parental conflict (Minuchin, 1974).

Mental illness in the family is the only dysfunctional family circumstance that showed a relation with the occurrence of ED, suggesting that we should not confine investigation to the ACE concept used here but look at a broader range of family circumstances.

## Strength and limits

The study investigated the ED risk associated with ten early intrafamilial ACEs and thus gives a differentiated account of trauma in the background of EDs.

No previous ED studies have been found in Central and Eastern Europe with a wide-ranging investigation into intrafamilial harm.

The regional setting may enhance the transcultural value of the study (e.g., relating to the role and recognition of family aggression).

We have confirmed the former Hungarian SCOFF data on ED risk in an adolescent population, and new data have been provided about the relation of ED risk and ACE.

Using a mixed sample (boys and girls), the study differs from most others, which have been largely or exclusively based on female samples, and have provided little data on mixed-gender samples and even less on male samples. The use of mixed and male samples is essential to determine whether the previously found differences between sexes are decreasing, or how they are changing.

A cross-sectional study limits the interpretability of data. Moreover, the assessment of the ACEs and the SCOFF was based on self-report, which may cause some bias. The ACE Score Calculator is a short retrospective 10-item screening tool, which may also lead to biased results.

The sample cannot be regarded representative, but as it includes adolescents from a wide range of social backgrounds, it can effectively cover the current situation.

Our results could be further refined with a larger sample, which would also be necessary to investigate the involvement of ACEs in other ED types (e.g., orthorexia nervosa).

## Future directions

An epidemiological study of other childhood and adolescent ED types (orthorexia, selective eating) is required, as is an evaluation of their association with ACEs.

Other forms of family dysfunction that may be involved in EDs (e.g., overprotection) should also be evaluated, including those that do not conform to the present concept of ACE.

## Conclusion

Our findings underline the importance of adolescent ED risks because prevention and early intervention in adolescence is of utmost importance.

The ED risk factors our study found to be most important were emotional, physical and sexual abuse, and emotional neglect. This is consistent with previous findings; in addition, our study found a new risk factor, namely a family member with a mental illness.

## Data availability statement

The raw data supporting the conclusions of this article will be made available by the authors, without undue reservation upon reasonable request.

## Author contributions

BK-T and FT: conception or design of the work. BK-T and BO: data collection. BK-T, BO, IK, and FT: data analysis and interpretation, critical revision of the article, and final approval of the version to be published. BK-T: drafting the article. All authors contributed to the article and approved the submitted version.

## Conflict of interest

The authors declare that the research was conducted in the absence of any commercial or financial relationships that could be construed as a potential conflict of interest.

## Publisher’s note

All claims expressed in this article are solely those of the authors and do not necessarily represent those of their affiliated organizations, or those of the publisher, the editors and the reviewers. Any product that may be evaluated in this article, or claim that may be made by its manufacturer, is not guaranteed or endorsed by the publisher.

## Appendix

The ACE score calculator—preambles, item contents and response options.

**Table:**

Item	Preamble and content	ACE category
	During your life:	
1a	Did a parent or other adult in the household often or very often … Swear at you, insult you, put you down, or humiliate you? or Act in a way that made you afraid that you might be physically hurt?	Emotional abuse
2a	Did a parent or other adult in the household often or very often … Push, grab, slap, or throw something at you? or Ever hit you so hard that you had marks or were injured?	Physical abuse
3a	Did an adult person at least 5 years older than you ever … Touch or fondle you or have you touch their body in a sexual way? or Attempt or actually have oral, anal, or vaginal intercourse with you?	Sexual abuse
4a	Did you often or very often feel that … No one in your family loved you or thought you were important or special? or Your family did not look out for each other, feel close to each other, or support each other?	Emotional neglect
5a	Did you often or very often feel that … You did not have enough to eat, had to wear dirty clothes, and had no one to protect you? or Your parents were too drunk or high to take care of you or take you to the doctor if you needed it?	Physical neglect
6a	Were your parents ever separated or divorced?	Parental separation/divorce
7a	Was your mother or stepmother: Often or very often pushed, grabbed, slapped, or had something thrown at her? or Sometimes, often, or very often kicked, bitten, hit with a fist, or hit with something hard? or Ever repeatedly hit for at least a few minutes or threatened with a gun or knife?	Witnessing violent treatment of mother
8a	Did you live with anyone who was a problem drinker or alcoholic or who used street drugs?	Household substance abuse
9a	Was a household member depressed or mentally ill, or did a household member attempt suicide?	Household mental illness
10a	Did a household member go to prison?	Incarcerated household member
	A dichotomous scale – yes/no	

## Abbreviations

ACEs: Adverse childhood experiences
AN: Anorexia nervosa
BMI: Body mass index
BN: Bulimia nervosa
EAT: Eating attitudes test
EDs: Eating disorders
EDI: Eating disorder inventory
